# Possible Aripiprazole-Induced Hypothermia: An “Icy” Side Effect?

**DOI:** 10.7759/cureus.19855

**Published:** 2021-11-24

**Authors:** Sambhawana Bhandari, Maun R Baral, Jorge Aguilar Zanatta

**Affiliations:** 1 Internal Medicine, Danbury Hospital/Nuvance Health, Danbury, USA; 2 Psychiatry, Nuvance Health, Danbury, USA

**Keywords:** antipsychotic side effect, aripiprazole, aripiprazole and hypothermia, temperature dysregulation by antipsychotics, hypothermia

## Abstract

Antipsychotics are a widely used class of drugs. They have been frequently associated with temperature dysregulation, especially hyperthermia. Hypothermia is also a rare but very serious side effect associated with these drugs. We present a case of possible aripiprazole-induced hypothermia with normothermia achieved after its discontinuation. An 81-year-old woman was brought into the emergency room with hypotension, hypothermia, and bradycardia. She was initially managed with intravenous fluids, external rewarming, and broad-spectrum antibiotics. Blood cultures and workup for infection returned negative with a low procalcitonin. Workup for endocrinopathy was also negative. She needed a Bair Hugger™ (3M, Maplewood, MN) to keep her temperature above 36 °C even after her other vital signs had normalized. Finally, her aripiprazole was held with the suspicion that it was causing hypothermia. Following this, her temperature improved and remained stable throughout even after discharge. Since hypothermia can be life-threatening, after ruling out common causes, clinicians should consider aripiprazole-induced hypothermia in these patients and the drug should be promptly discontinued.

## Introduction

Hypothermia is defined as a drop in core body temperature to levels below 35 °C or 95 °F [1]. The presentation of hypothermia can range from shivering to hypotension, bradycardia, coma, and even death [2]. Hypothermia can be caused by various factors, including low temperatures, hypothyroidism, diabetic ketoacidosis, sepsis, and prolonged cardiac arrest, to name a few [1,2]. Some medications are also known to cause hypothermia and multiple antipsychotics have been associated with temperature dysregulation [3-5]. The better-known association however is with hyperthermia (in relation to neuroleptic syndrome) rather than hypothermia. Hypothermia has been observed as starting within two to seven days after initiating or a recent increase in the dose of an antipsychotic; however, there have also been cases involving patients who were on stable doses of antipsychotics for a long time [3,6].

## Case presentation

An 81-year-old woman living in a long-term care facility presented with complaints of dizziness. Her medical history included schizophrenia, hypothyroidism, hyperlipidemia, transient ischemic attack, and hypertension. Medications at home were as follows: aripiprazole 5 mg once daily, fluoxetine 20 mg once daily, clopidogrel 75 mg daily, levothyroxine 25 mcg in the morning, amlodipine 10 mg daily, and lisinopril 20 mg daily. On presentation, it was found that she was hypotensive with a blood pressure of 94/63 mmHg, bradycardic with a heart rate of 50/minute, hypothermic with a rectal temperature of 31.5 °C, and SpO_2_ of 96% on room air.

Her laboratory workup was relevant for blood urine nitrogen (BUN)/creatinine of 40 mg/dL/2.79 mg/dL, and urine toxicology was positive for opiates and benzodiazepines. There was no leucocytosis; her white blood cell count was 4.2 x 10^9^/L, hemoglobin was 10.7 g/dL, and platelets were 142 x 10^9^/L. Her activated partial thromboplastin time (APTT) was 42, prothrombin time (PT) was 13.6 seconds, and the international normalized ratio (INR) was 1.0, with chemistry showing normal electrolytes. There was no lactic acidosis and troponin was undetectable. Thyroid-stimulating hormone (TSH) was checked due to a suspicion of endocrinopathy, mainly hypothyroidism/myxedema, which returned normal (1.53 mIU/L).

CT scan of the head was unremarkable. Electrocardiography was suggestive of first-degree heart block. Initially, a septic shock protocol with intravenous fluids and external rewarming for hypothermia was started. Intravenous levofloxacin and Flagyl were started after taking the blood sample for culture and sensitivity; 100 mg of hydrocortisone was administered intravenously for suspected adrenal insufficiency. Despite these measures, the patient continued to be hypotensive. Hence, she was started on a dopamine drip, which led to rapid improvement. It was then gradually titrated and stopped once the blood pressure was stable. Blood cultures were negative and the procalcitonin level was 0.07 ng/ml. Hence, her antibiotics were discontinued.

Although her other vital signs improved, her temperature did not. She was placed on a Bair Hugger™ (3M, Maplewood, MN) to keep her temperature above 36 °C. In evaluating the causes of her hypothermia, it was suspected that her antipsychotics could be responsible. We discontinued them after consulting Psychiatry during the hospital admission. Workups consistently remained negative for any septic etiology or endocrinopathy, namely thyroid and adrenal. Her acute kidney injury resolved after fluid administration and was thought to be due to a prerenal cause. It was not clear why her urine toxicology was positive for benzodiazepines and opiates. When we called the facility, we were told that they managed her medications themselves and that there were no benzodiazepines or opiates on the list.

Hence, aripiprazole was held with suspicion of hypothermia even though there had been no dose adjustments according to her caretaker at the facility where she lived. Her temperature improved and remained stable without the need for the Bair Hugger™ after the discontinuation of aripiprazole. Thus, it was held upon discharge. After discharge, the facility was called to confirm her body temperature, which was noted to be normal on multiple occasions.

## Discussion

Hypothermia is defined as a drop in core body temperature to levels below 35 °C or 95 °F [1]. Hypothermia can result from heat loss, abnormalities of heat conservation or production, or failure of central thermoregulation [6]. Hypothermia can be caused by various factors, such as low temperatures, hypothyroidism, diabetic ketoacidosis, sepsis, and prolonged cardiac arrest, to name a few [1,2]. There can be a combination of the above causes in any clinical scenario. Table 1 illustrates the classifications of hypothermia.

**Table 1:** Classification of hypothermia* *[1,2,6]

Grade	Range of core temperature	Symptoms and signs
Mild	90-95 °F or 33-35 °C	Vigorous shivering, cold diuresis, cold white skin
Moderate	82-90 °F or 28-33 °C	Reduced shivering, slurred speech, amnesia, confusion, apathy, hyporeflexia, ataxia, bradycardia
Severe	Less than 82 °F or 28 °C	Loss of shivering, bradycardia, suppression of cardiac systolic function, hypotension, hypoventilation, cardiac arrest, fixed dilated pupils, and death

Body temperature is regulated by the preoptic anterior hypothalamus with the involvement of dopamine, serotonin, norepinephrine, and α-adrenergic receptors. Aripiprazole is a psychotropic drug with partial agonist activity at D2 receptor in the mesolimbic pathway and in the mesocortical pathway, and 5-HT1A receptors with potent antagonism at 5-HT2A receptors [7]. As the stimulation of the 5-HT1 receptor results in a decrease in body temperature and stimulation of the 5-HT2 receptor leads to an increase, it is hypothesized that antagonism of the 5-HT2 receptor by atypical antipsychotics may be the reason for hypothermia associated with their use [6,7]. It has been mostly observed to start within two to seven days of the administration or a recent dose increase; however, there have also been cases where there were no changes made in a stable, long-term dose or were largely unknown [3,6].

Our patient had been taking aripiprazole chronically without any recent dose adjustments. Therefore, initially, we did not consider antipsychotic-induced hypothermia in our differentials. However, blood cultures and procalcitonin as well as chest X-ray and urinalysis were negative, thereby ruling out most of the infectious etiologies. Antibiotics were also discontinued after that. Her cortisol and TSH levels were normal. Her facility denied any exposure to cold or the use of any other drugs such as benzodiazepine, which could have led to synergistic effects.

Most importantly, the patient's hypothermia persisted even after other vital signs had normalized. Moreover, she was normothermic after the medication (aripiprazole) was discontinued. She was stable on the day of the discharge and at multiple follow-ups after discharge.

## Conclusions

Antipsychotics have been associated with temperature dysregulation, among which hyperthermia is a better-known side effect. Hypothermia is a rare but life-threatening side effect associated with these types of medications. Although our case had many associated factors that could have contributed to hypothermia, the onset of normothermia upon the prompt discontinuation of the drug leads us to believe that it was probably associated with the use of aripiprazole.

In circumstances like this, drug-induced hypothermia should be considered after ruling out other more common causes. The possible offending agent should be immediately discontinued.
